# The Stem Cell Marker CD133 Associates with Enhanced Colony Formation and Cell Motility in Colorectal Cancer

**DOI:** 10.1371/journal.pone.0010714

**Published:** 2010-05-19

**Authors:** Tarek M. A. Elsaba, Luisa Martinez-Pomares, Adrian R. Robins, Simon Crook, Rashmi Seth, Darryl Jackson, Amy McCart, Andrew R. Silver, Ian P. M. Tomlinson, Mohammad Ilyas

**Affiliations:** 1 Division of Pathology, School of Molecular Medical Sciences, Queen's Medical Centre, University of Nottingham, Nottingham, United Kingdom; 2 Department of Pathology, South Egypt Cancer Institute, Assiut University, Assiut, Egypt; 3 Institute of Infection, Immunity and Inflammation, School of Molecular Medical Sciences, Queen's Medical Centre, University of Nottingham, Nottingham, United Kingdom; 4 Centre for Academic Surgery, Institute of Cell and Molecular Science, Barts and The London School of Medicine and Dentistry, London, United Kingdom; 5 Molecular and Population Genetics, Wellcome Trust Centre for Human Genetics, University of Oxford, Oxford, United Kingdom; 6 Nottingham Digestive Diseases Centre–Biological Research Unit, Queen's Medical Centre, Nottingham, United Kingdom; Roswell Park Cancer Institute, United States of America

## Abstract

CD133 is a membrane molecule that has been, controversially, reported as a CSC marker in colorectal cancer (CRC). In this study, we sought to clarify the expression and role of CD133 in CRC. Initially the size of the CD133−expressing (CD133+) population in eight well-described CRC cell lines was measured by flow cytometry and was found to range from 0% to >95%. The cell line HT29 has a CD133+ population of >95% and was chosen for functional evaluation of CD133 after gene knockdown by RNA interference. A time course assay showed that CD133 inhibition had no significant effect on cell proliferation or apoptosis. However, CD133 knockdown did result in greater susceptibility to staurosporine-induced apoptosis (p = 0.01) and reduction in cell motility (p<0.04). Since gene knockdown may cause “off-target” effects, the cell line SW480 (which has a CD133+ population of 40%) was sorted into pure CD133+ and CD133− populations to allow functional comparison of isogenic populations separated only by CD133 expression. In concordance with the knockdown experiments, a time course assay showed no significant proliferative differences between the CD133+/CD133− populations. Also greater resistance to staurosporine-induced apoptosis (p = 0.008), greater cell motility (p = 0.03) and greater colony forming efficiency was seen in the CD133+ population than the CD133− population in both 2D and 3D culture (p<0.0001 and p<0.003 respectively). Finally, the plasticity of CD133 expression in tumour cells was tested. Quantitative PCR analysis showed there was transcriptional repression in the CD133− population of SW480. Prolonged culture of a pure CD133− population resulted in re-emergence of CD133+ cells. We conclude that CD133 expression in CRCs is associated with some features attributable to stemness and that there is plasticity of CD133 expression. Further studies are necessary to delineate the mechanistic basis of these features.

## Introduction

Recent years have seen the emergence of the “cancer stem cell hypothesis” which postulates that a minority population of cells within a tumour consists of cancer stem cells (CSC) [1], [2]. This population is purportedly responsible for generating the bulk of the tumour which consists of cells in varying degrees of differentiation. The hierarchy of a tumour is thus thought to be similar to the tissue from which the tumour originates and CSCs are deemed neoplastic counterparts of stem cells in the normal tissue. In this respect, CSCs would be expected to have a stem cell-like phenotype (generally referred to as “stemness”). This is characterised by features such as limitless replicative ability, multipotency and resistance to apoptosis [3]. The stem cell phenotype may also include cyto-protective strategies such as ability to actively extrude dangerous substances from the cell–a feature which may be the basis of resistance to chemotherapeutic agents [3], [4].

In parallel with the emergence of the cancer stem cell hypothesis, there has been a growing interest in the isolation and study of CSCs. A number of studies claim to have isolated CSCs from several different tumour types such as brain [5], [6], breast [7], colon [8], [9], hepatocellular carcinoma [10] and pancreatic cancer [11]. These studies have used putative CSC markers to separate stem cells from differentiating cells within a tumour. One common method of separation is the dye elimination method (i.e. side population [12]) although identification of a number of cell surface markers (such as CD24, CD44, CD166, and integrins) has allowed use of fluorescence activated cell sorting (FACS) to isolate CSCs [13]. One marker consistently reported as a stem cell marker in tumours of differing origins is CD133 (also known as Prominin 1).

The CD133 gene (*PROM1*) maps to chromosome 4p15 and encodes a 120 kD transmembrane glycoprotein (ENSG00000007062). The CD133 protein is pentaspan cell surface receptor although neither its ligand nor its secondary messengers are known. It was first recognized as a surface marker for haematopoietic stem cells [14], [15]. Later, it was used to recognize cancer stem cells in many solid tumours arising in, for example, breast [13], pancreas [16] and liver [17].

Two recent studies identified CD133 as a marker for stem cells in colorectal cancers (CRC) [8], [9]. In these studies, CD133 expressing (CD133+) cells were isolated from primary CRCs and were shown to have the ability to form tumours as xenografts in nude mice; in contrast CD133− cells from the same tumours were shown to have very limited ability to form xenografts and even that was attributed to contamination by CD133+ cells. Analysis of the xenografts that were formed showed that the size of CD133+ population was similar to that seen in the primary tumour and, furthermore, the ability to continuously propagate the xenografts was tightly associated with CD133 expression. Subsequent studies, however, have not validated these observations [18] and in one study it has been reported that, in fact, the CD133− population has the greater colony forming ability [19]. Our study sought to further clarify the expression and the role of CD133 in CRC. Two approaches were used: (i) CD133 expression was functionally evaluated in HT29 after gene knockdown and (ii) SW480 underwent cell sorting into a CD133+ population and a CD133− population followed by comparative functional analysis of the two populations.

## Materials and Methods

### Tissue culture, flow cytometry and fluorescence activated cell sorting

Eight human colorectal cancer cell lines (Caco2, DLD1, HT29, Lovo, LS1034, SW480, SW620, SW837) were maintained as previously described [20]. Identity of all cell lines was confirmed by fingerprinting for mutations in *KRAS, BRAF, PI3KCA, CDC4, TP53* and testing microsatellite instability.

Evaluation of the size of the CD133 expressing (CD133+) population in each cell line was undertaken by flow cytometry using a phycoerythrin (PE) labelled antibody - CD133/1 (clone AC133/1, Miltenyi Biotec, UK). Cells were detached using non-enzymatic cell dissociation solution (Sigma) and approximately 5×10^5^ cells were incubated with antibody (diluted 1∶100 in FACS wash (0.5% bovine serum albumin; 2 mM NaN_3_; 5 mM EDTA)) for 15 minutes at 4°C. An isotype and concentration matched PE labelled control antibody (Miltenyi Biotec, UK) was used and samples labelled with this antibody were used to set the gating levels. After three 5 minute washes with FACS wash, the cells were re-suspended and fixed in solution containing FACS wash with 1% formaldehyde. Determination of percentage of CD133+ cells and sorting of cell lines into CD133+/CD133− populations were performed on an Epics Altra flow cytometry machine (Beckman Coulter). The results were analyzed using WinMDi 2.9 computer software.

For fluorescence activated cell sorting (FACS) of SW480, the cells were labelled using the same protocol but without the final fixation step. In order to evaluate plasticity of CD133 expression, sorted cells were maintained in culture for 3 weeks before undergoing re-evaluation. For all other experiments, the CD133+/CD133− populations were tested immediately after sorting.

### RNA extraction, detection of splice variants and mRNA quantification

Total RNA was extracted from cells using the RNeasy Mini Kit (Qiagen) following the manufacturer's instructions. Samples of normal colonic mucosa were collected with full approval of the local ethics committee (Oxford Research Ethics committee B, REC reference C02.310). Written informed consent was obtained from all patients whose samples were tested for CD133 expression and splice variants. RNA was extracted and complementary DNA (cDNA) was synthesized as previously described [20].

Two main splice variants have been described for CD133. The larger variant AC133-1 (NM_006017) was identified first and contains all exons [15]. The second variant, AC133-2, splices out exon 4 which is 27 base pairs long and encodes a 9 amino acid sequence in the first extracellular domain of CD133. Expression of splice variants was tested by RT-PCR using an upstream primer in exon 3 and a downstream primer in exon 5 (primer sequences available from the authors). This would produce a product of 199 base pairs from AC133-1 and 172 bp from AC133-2. End point PCR analysis was performed by viewing PCR products on agarose gels. However, given that preferential amplification of the smaller product may produce data artefacts, the study was repeated using real-time PCR (with Sybr green as the reporter dye) on the MX3005P thermal cycler (Stratagene, UK). Specificity of the PCR was validated by bidirectional direct sequencing. The presence of splice variants was tested in cDNA obtained from the CRC cell lines, the sorted CD133+ and CD133− populations of SW480 as well as 10 samples of normal colonic mucosa

Sorted CD133+/− populations underwent analysis of mRNA levels by quantitative RT-PCR analysis using the standard curve method. Analysis was performed in triplicate for each sample and *CD133* values were normalized to the housekeeping gene *HPRT*. Each reaction was performed in a final volume of 25 µl in a MX3005P thermal cycler (Stratagene, UK). The data for Q-PCR were analyzed using the MxPro-QPCR software.

### Gene knockdown

Gene knockdown was achieved by transfecting HT29 cells (shown to have high CD133 expression) with CD133−specific small interfering RNA (siRNAs). Approximately 2×10^5^ cells were seeded in 2 ml DMEM media with 10% FBS in 6 well plates (Corning) 24 hours prior to transfection. Cells were transfected with CD133-specific stealth siRNA duplexes (sequence: 5′-GAGUCGGAAACUGGCAGAUAGCAAU-3′, Invitrogen) at a final concentration of 33 nM using Lipofectamine 2000 (Invitrogen) in accordance with the manufacturer's instructions. These are henceforth annotated as HT29^CD133−^. Controls consisted of cells transfected with scrambled control siRNA (sequence: 5′-GAGGGAACAGUCGGAUAGACCUAAU-3′) and are henceforth annotated as HT29^ssc^ (scrambled siRNA control).

### Western blotting

For Western blotting, lysates were prepared and quantified as previously described [20]. Samples (30 µg) were subjected to Sodium Dodecyl Sulfate Polyacrylamide Gel Electrophoresis (SDS-PAGE) using a 10% resolving polyacrylamide gel and transferred onto a Hybond-P PVDF membrane (Amersham Bioscience). After blocking with 5% Bovine Serum Albumin (BSA)/0.1% TPBS (Tween20 in PBS solution) for 60 minutes, the membrane was then incubated over night at room temperature with CD133 (C24B9) Rabbit mAB (Cell Signaling) in a concentration of 1∶1000. The membrane was washed with 0.1% TPBS 3 times for 5 minutes each then incubated for 1 h at room temperature with a horseradish peroxidase-linked secondary antibody (Sigma Aldrich) (1∶10000, anti-rabbit IgG) diluted with 5% BSA/PBS containing 0.1% Tween20. After a further 3 washes, the membrane was visualized enhanced chemiluminescence reagents (Thermo scientific) For a loading control, the monoclonal Anti-β-actin antibody (Sigma Aldrich) in a dilution of 1∶2000 against the β-actin protein was used.

### Proliferation assays

A time course assay for proliferation was performed after gene knockdown and after cell sorting. For HT29, each well of a 24 well plate was seeded with 10^4^ cells 24 hours after transfection with either CD133 specific or scrambled control siRNA. Cell numbers were assessed on days 1, 3 and 5. For SW480 the sorted CD133+ and CD133− populations were seeded at 10^4^ cells per well and cell numbers assessed on days 1, 3, 5, 7 and 9. At least two independent experiments, each in triplicate, were performed. A methylene blue assay [21], [22] was used to quantify numbers of adherent viable cells.

### Stauropsorine induced apoptosis

Staurosporine was used to evaluate the resistance conferred by CD133 to exogenous apoptotic stresses. For HT29, each well of a 96 well plate (Costar) was seeded with either HT29^CD133−^ or HT29^ssc^ cells 72 hours after transfection. Approximately 5×10^4^ cells were seeded per well and incubated with staurosporine (Sigma) at a final concentration of 8 µM for 24 hours. After this period, viable and apoptotic cells were measured. For SW480, each well of a 96 well plate was seeded with 10^5^ cells of the sorted cells and staurosporine was added 24 hours later at a concentration of 8 µM. After another 24 hours, the numbers of viable cells/apoptotic bodies were assessed. The assay was performed in triplicate and repeated in at least two independent experiments.

### 2 dimensional (2D) and 3 (3D) dimensional colony forming assay

The ability of isolated single CD133+ and CD133− cells to form colonies was tested in both 2 dimensional (2D) culture and 3 dimensional (3D) culture. For 2D culture, 300 freshly sorted cells were seeded into individual wells of a 6 well plate and cultured for 14 days. The cells were then stained with methylene blue and colonies containing more than 20 cells were counted. The experiments were carried out in triplicate and on two occasions.

3D culture was performed to assess the colonogenic ability of the sorted populations in non adherent conditions. Cells (2500 from each population CD133+ and CD133-cells) were counted and resuspended as single cells in 0.7% DNA grade agarose (Sigma Aldrich).This was overlaid on a base of 1% DNA grade agar (Sigma Aldrich) and both top and base layers were mixed with 2X DMEM. Experiments were set up in triplicate and medium changed twice a week. After two weeks, the number of colonies that developed within each well was counted and visualized under a microscope after staining with 0.05% crystal violet for 1 hour and representative fields were photographed. For both 3D and 2D culture, percent colony forming efficiency (CFE) was calculated as follow, % CFE  =  (Number of obtained colonies/Number of cultured cells) X 100 [23]. Log transformed values were then used for statistical analysis.

### *In vitro* migration assay

Transwell cell migration assays were performed using a Boyden chamber containing a polycarbonate filter with an 8 µm pore size (Costar). Culture medium (600 µl) supplemented with 20% FBS was added to the lower chamber and 2.5×10^5^ cells of the sorted populations were added into the upper chamber (in 100 µl of culture medium supplemented with 10% FBS). The number of cells migrating through the membrane was manually counted after 24 hours. Assays were performed in triplicate and on two separate occasions. Cell migration was also measured using a cell wounding assay performed in 6 well plates (Costar). Cells were grown to confluence and then starved for 24 hours in serum free medium. A sterile 200 µl pipette tip was used to create three separate parallel wounds and migration of the cells across the wound line was assessed after 24 hours. Photographs were taken using a charge-coupled device (CCD) camera (Canon, Japan) attached to the inverted phase-contrast microscope at a power of X40. The distance between the edges was measured at 6 equally distributed points using ImageJ software [33] and then analysed using a two tailed t-test. Experiments were repeated on two separate occasions.

### Statistical analysis

Statistical analysis was undertaken using SPSS 13.0 Software. All evaluations were done using unpaired two tailed Student's T-test. For studies involving cell and colony counting, cell numbers were analysed. For studies with a numerical fluorescence output, the raw fluorescence values were used.

## Results

### CD133 expressing populations in cell lines

**Figure 1:**
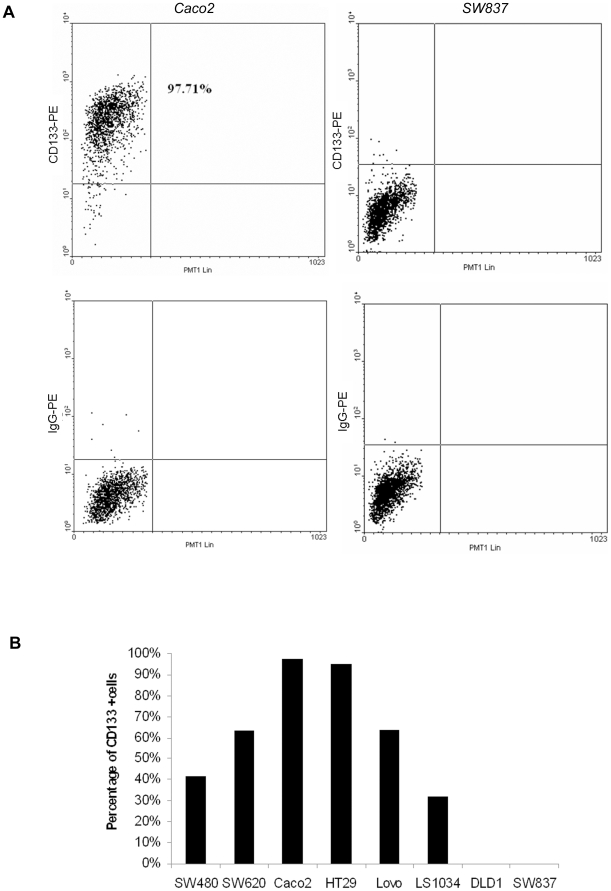
Evaluation of CD133+ populations by flow cytometry. Eight CRC cell lines were tested and showed varying size of the population of CD133+ cells. (a) Sample flow cytometry images are shown for Caco2 (left panels, >95% positive cells) and SW837 (right panels). Analysis was performed using AC133/1 antibody (top panel) and gating was performed for each cell line using an isotype control (bottom panel), PMTLin 1 is photmultiplier tube linear 1 which indicates side scatter) (b) shows that in two cell lines there were no CD133+ cells identified whilst in two cell lines nearly all cells were CD133+. The remaining cell lines had varying size population of CD133+ cells.

The size of the CD133+ population was tested in 8 CRC cell lines by flow cytometry and in each case approximately 500 000 cells were analysed. In two cell lines (DLD1 and SW837), CD133+ cells were not detectable. The size of CD133+ population was found to be >95% in two cell lines (Caco2 and HT29). In the remaining cell lines, a bimodal population was present with a CD133+ population ranging from 32–64% (Figure 1).

### Splice variants of CD133 in CRC cell lines and normal mucosa

**Figure 2:**
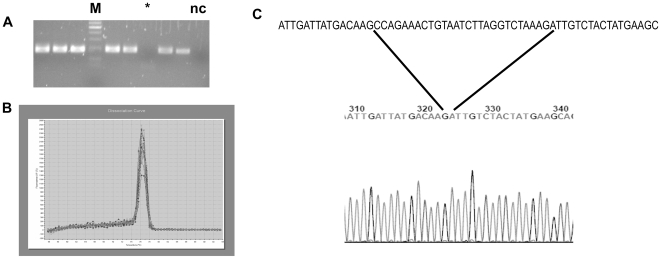
Splice variants of CD133. Cell lines and normal mucosal samples were tested for expression of the two main CD133 splice variants by RT-PCR with primers anchored on either side of the spliced out exon (exon 4). Sample data are shown which demonstrate only a single product was identified when PCR products were analysed on both (a) agarose gel and (b) the more sensitive Q-PCR technique using Sybr green as a reporter. M  =  size marker, nc  =  negative control, * represents DLD1. (c) Sequencing of the products showed that exon 4 was spliced out of the coding sequence thus only shorter splice variant (AC133-2) was being expressed. The sequence above the electropherogram is of AC133-1 with exon 4 shown between the lines.

Expression of the two main splice variants of CD133 (AC133-1 and AC133-2) was tested by RT-PCR in both the cell lines and samples of normal mucosa. Only one product was seen on agarose gels which, on sequencing, was found to be the shorter AC133-2 splice variant from which exon 4 is spliced out (Figure 2a and 2c). However, shorter PCR products undergo preferential amplification and it is possible that larger products may be missed by both agarose gel electrophoresis and sequencing (especially if present at low levels). The analysis was refined by performing PCR using SYBR green as a reporter dye. Evaluation of the dissociation curve showed the presence of a single PCR product only (Figure 2b).

### Knockdown of CD133

**Figure 3:**
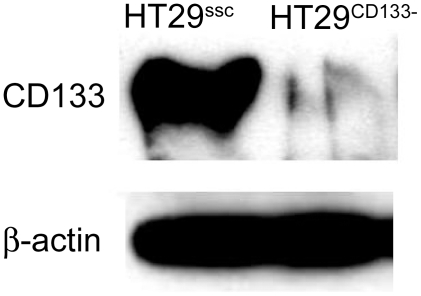
Knockdown of CD133. HT29 cells were transfected with either CD133 specific siRNA (HT29^CD133−^) or scrambled control ((HT29^ssc^). Western blotting confirmed the loss of CD133 expression by gene knockdown. β-Actin shows equal protein loading.

HT29 has a high level of CD133 expression. Cells were transfected with either CD133 specific or scrambled control siRNA and were tested 72 hours post transfection. Western blot showed that the protein was virtually undetectable in the when CD133 specific siRNA was used. In contrast, transfection of the control siRNA has no effect on protein expression (Figure 3).

### Association of CD133 expression with cell proliferation

**Figure 4:**
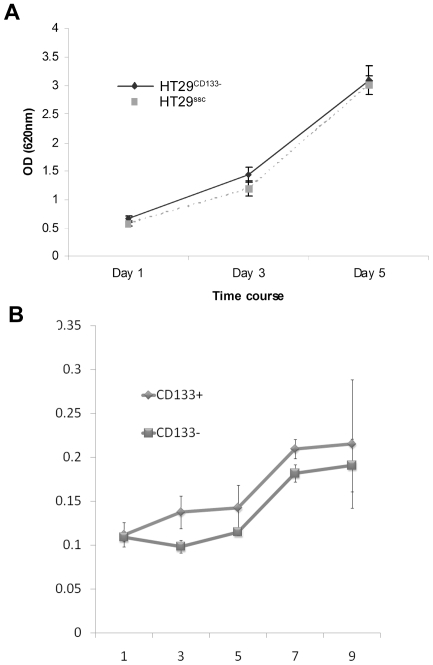
Effect of CD133 on proliferation. Cell proliferation was evaluated after knockdown of CD133 in HT29 (Figure 4a) and sorting of SW480 into pure CD133+ and CD133− populations (Figure 4b). A time course assay was performed over several days with no association seen between CD133 and cell numbers.

We compared the proliferation rate between cells with high and low CD133 levels using two experimental approaches. In HT29 the CD133 protein was knocked down and cell numbers monitored for 5 days (Figure 4a) whilst SW480 was sorted into CD133+/CD133− populations and cell numbers were monitored over 9 days (Figure 4b). Both experiments showed the same results i.e. there was no significant difference between cells with high or low CD133 expression. Similarly, counts of floating apoptotic bodies over this period did not show any difference (data not shown).

### Association of CD133 expression with staurosporine induced apoptosis and colony formation

**Figure 5:**
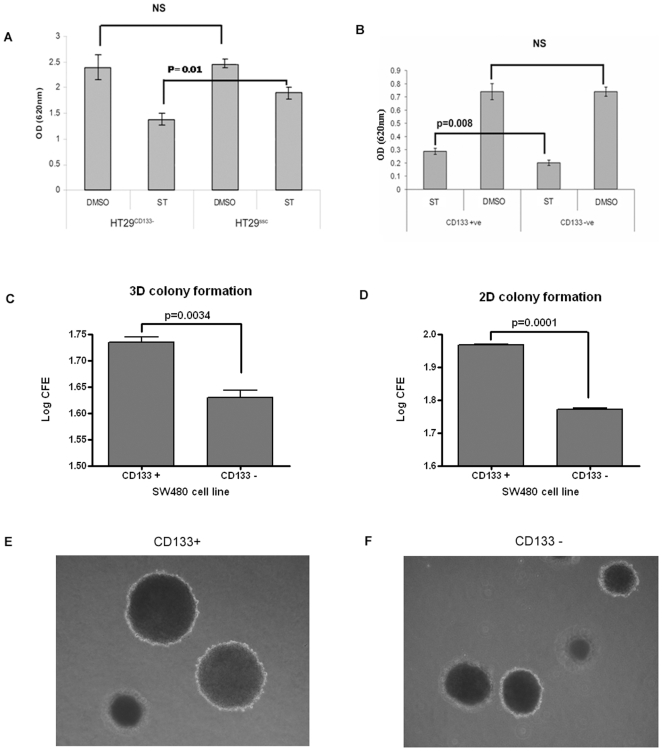
Association of CD133 expression with features of stemness. CD133 gave resistance to staurosporine induced apoptosis. Figure 5a shows that after exposure to staurosporine there were fewer viable cells after transfection with CD133 specific siRNA (HT29^CD133−^) than with scrambled control (HT29^ssc^) (p = 0.01). There were no differences between the cells after exposure to DMSO. Figure 5b shows that the CD133+ population of SW480 showed cells showed significantly greater resistance than the CD133− population (p = 0.008). Figure 5c and 5d show CD133+ cells were significantly more clonogenic than CD133− cells (p<0.0001 and p<0.003) in 2D and 3D culture respectively. Figures 5e (colonies formed by CD133+ cells) and 5F (colonies formed by CD133− cells) show that both CD133+ and CD133− populations of SW480 were able to form colonies from single cells (typical colonies are shown).

A feature that may be expected in stem cells is resistance to apoptosis following exogenous stress. Both experimental approaches were used to test the effect of CD133 levels on resistance to apoptosis when exposed to staurosporine for 24 hours. Concordant results were obtained indicating that high levels of CD133 conferred staurosporine resistance. Fewer viable HT29^CD133−^ cells were present than HT29^ssc^ cells (Figure 5a, p = 0.01); conversely greater number of viable cells were present in the sorted CD133+ population than the CD133− population(Fig. 5b, p = 0.008). There was however no difference between cells when exposed to DMSO alone.

Another features of “stemness” is the ability to form colonies. This was tested by growing single cells in soft agar for two weeks (3D culture) and 2D culture and the sorted CD133 + and CD133 − cells were compared. In both cases, colonies were visible after two weeks but the number of colonies was significantly greater in CD133 + cells (P<0.0001, and P<0.003) in 2D and 3D culture respectively (Figure 5c and 5d).

### Association of CD133 expression with cell migration

**Figure 6:**
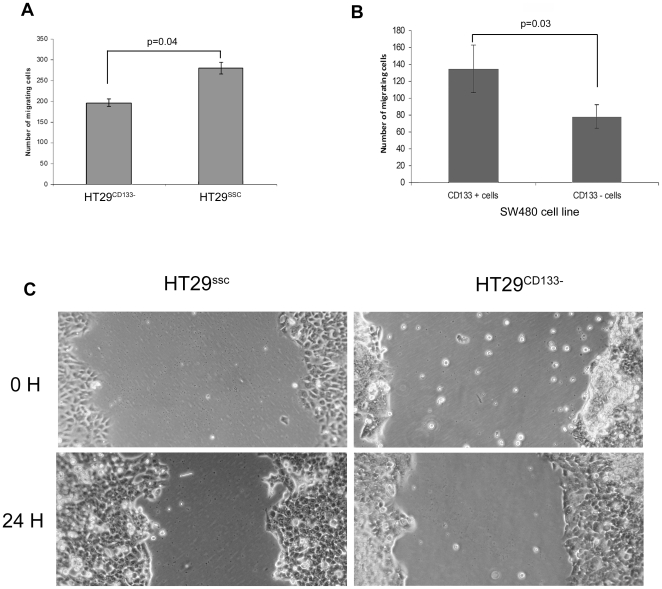
Effect of CD133 on cell motility. Transwell migration was measured after knockdown of CD133 in HT29 and sorting of SW480 into CD133+/− populations. Significantly fewer HT29^CD133−^ cells migrated across the membrane than HT29^ssc^ cells (Figure 6a, p = 0.04). Conversely, larger numbers of sorted CD133+ cells migrated than CD133− cells (Figure 6b, p = 0.03). A wounding assay was also undertaken and gene knockdown was associated with marked delay in closure of the wound which was visiually perceptible after only 24 hours (Figure 6c) and statistically significant (p<0.001).

Comparative analysis of cell motility between cells with high and low CD133 expression was tested by transwell migration assays. Both experimental conditions produced concordant results. Significantly fewer HT29^CD133−^ cells migrated across the membrane than HT29^ssc^ cells (Figure 6a, p = 0.04). Conversely, significantly larger numbers of CD133+ cells migrated than CD133− cells (Figure 6b, p = 0.03). A cell wounding assay was also used following gene knockdown which showed that the wound edges were significantly closer in HT29^ssc^ cells than in HT29^CD133−^ cells (p<0.001) thereby confirming the relationship between high CD133 expression and increased cell motility (Figure 6c).

### Plasticity of CD133 expression in cell lines

**Figure 7:**
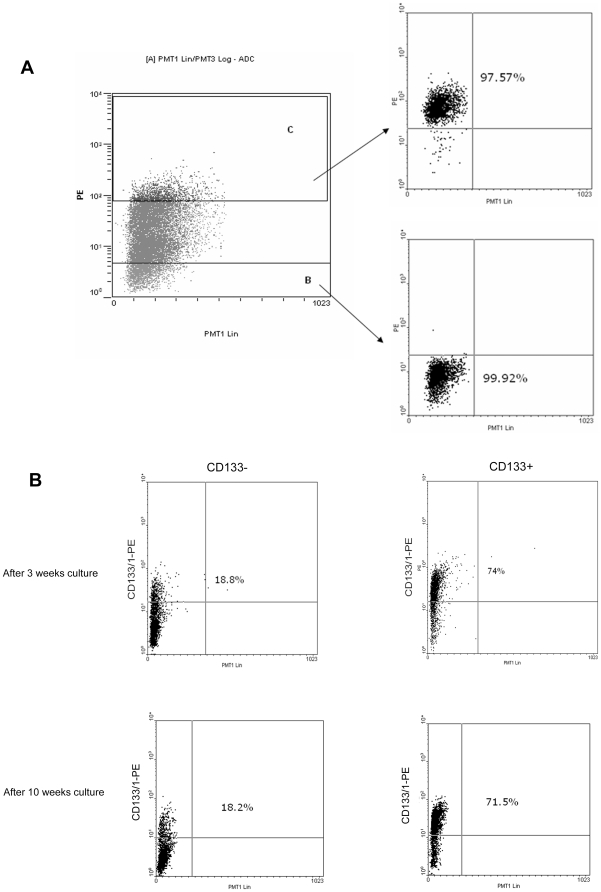
Plasticity of CD133 expression. SW480 was sorted into CD133 positive and negative populations which were then cultured separately. (a) Gating was set so that only the extreme populations were collected which, on immediate retesting, were shown to be very pure populations. (b) After prolonged culture, both of the sorted populations became biphasic. The ratios of the CD133+ and CD133− cells became stable after three weeks and did not change after that (although they were not the same as the original parental cell line).

**Figure 8:**
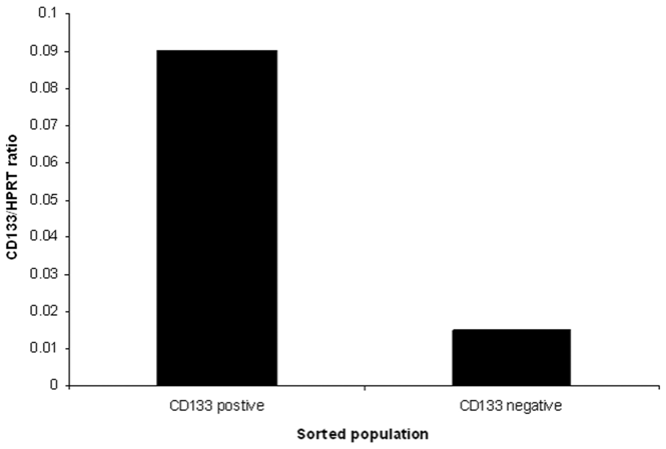
mRNA level in sorted populations. Q-RT-PCR analysis of the sorted populations showed that there was transcriptional repression of *CD133* in the CD133− population (although low levels of mRNA were still detectable).

The SW480 cell line was sorted into CD133+ and CD133− populations which were maintained in standard tissue culture conditions and underwent regular analysis by flow cytometry. Immediate re-analysis showed that the CD133+ and CD133− populations were, respectively, 97.6% and 99.9% pure (Figure 7a). Quantitative PCR showed transcriptional repression of CD133 in the CD133− population and although CD133 mRNA was still detectable, it was only at 15% of the level seen in the CD133+ population (Figure 8).

Prolonged culture resulted in both populations reverting to a bimodal profile. The CD133+ population, after 3 weeks, consisted of 70% CD133+ cells and 30% CD133− cells, frequencies which remained stable thereafter for at least 3 months. The findings run counter to published reports in which pure populations of CD133+ cells revert to normal ratio of CD133+/− populations [8]. The CD133− cells developed a population of CD133+ cells which, after 3 weeks, reached 17% but did not increase thereafter (Figure 7b).

## Discussion

### The CD133+ population is variable in CRC cell lines

We screened large numbers of cells from eight well established CRC cell lines by flow cytometry to quantify the size of the CD133+ population. We found that, in two cell lines (DLD1 and SW837) there were no detectable CD133+ cells, in two cell lines (Caco2 and HT29) the CD133+ population was >95% of the total tumour cells whilst the remaining cell lines had population sizes ranging from 32%–64%. Our data are in agreement with studies by letaet al. and Dalerba et al. [18], [24] who reported similar levels of CD133 expression in the cell lines HT29, Lovo and no expression in DLD1. Our data are however discrepant with those of O'Brien and Ricci-Vitiani [8], [9], although the cause of this discrepancy is unknown. One possibility is that our studies used established cell lines whereas the other studies used novel cell lines derived in their own laboratories. It is possible that prolonged culture may cause maintained changes in CD133 regulation although this in itself would suggest that CD133 expression is not a very good marker of CSCs. Differences in technique may provide another explanation although we used the same antibodies as O'Brien and Ricci-Vitiani and we kept incubation times with the primary antibody down to 15 minutes. Either way, our data have unequivocally shown that in 4/8 cell lines, the CD133+ population is either absent or comprises nearly the whole tumour cell population. These data together with other data presented below, lead us to conclude that CD133 is probably not a specific marker of stem cells in CRC.

### Only CD133 splice variant AC133-2 is expressed in colonic tissue

Although a wide variety of splice variants have been described for CD133, only one (termed AC133-1 and which was first to be cloned) has been allocated a Reference Sequence number. This contains the full length coding sequence whilst a second splice variant (AC133-2, the second to be cloned) appears to splice out exon 4. Our analysis of 8 CRC cell lines and 10 samples of normal mucosa, using both end-point and real-time methodologies, identified only variant AC133-2. This would be consistent with the study by Yu et al. [25] in which AC133-2 was reported as being the splice variant which is present in many stem cell compartments whilst AC133-1 is limited to foetal brain and skeletal muscle.

### CD133 expression is associated with some features of the stem cell phenotype

In order to evaluate the function of CD133, the cell line HT29 was tested after knockdown of CD133 by RNA interference. This method can also produce “off-target” effects and so a complementary approach was used to separate SW480–a cell line with a CD133+ population of 40% - into pure CD133+ and CD133− populations. Both types of experiments yielded similar results. Firstly, levels of CD133 did not appear to alter cell proliferation or apoptosis. There were however significant differences in the features which may be regarded as part of the stem cell phenotype. Thus high levels of CD133 were associated with increased clonogenicity and resistance to staurosporine induced apoptosis. The latter may be due to an innate resistance to apoptosis (possibly mediated by such reported associations as between CD133 expression and anti-apoptotic genes such as FLIP (Caspase 8 inhibitor) and Bcl-2 [26]. Alternatively, it may be due to enhanced cytoprotective strategies such as the ability to actively extrude toxic substances from the cytoplasm [3].

Another feature we found to be associated with CD133 expression was enhanced cell motility. Other studies have suggested a role for CD133 in cell motility since it is classically expressed in membrane protrusions [27], [28]. In certain situations, such as embryogenesis and wound healing, stem cells need to acquire features of motility. In CSCs, features of enhanced motility may allow invasion and metastasis to occur–a notion supported by the study of Rappa et al which found CD133 expression was associated with metastasis in melanoma cells [29]. Our data do, however, contradict those of Horst et al. [30] who did not find that CD133 expression was associated with cell motility in Caco2. We are uncertain of the cause for this discrepancy although this may be due to technical differences between laboratories, differences in cell lines or differences in the duplex sequence used for gene knockdown.

### CD133 shows plasticity of expression in CRC cell lines

We used FACS to sort SW480 into pure CD133+ and CD133− populations and, by mRNA quantification, showed that this was due to transcriptional repression rather than changes in glycosylation. The mechanistic basis of inhibiting CD133 expression is uncertain. Epigenetic silencing due to hypermethylation of CD133 promoter has been reported as a means of controlling CD133 expression [31]. Alternatively other mechanisms such as mRNA degradation may be involved. Prolonged culture of pure CD133+/CD133− populations resulted in both populations reverting to a bimodal pattern although in neither case did the pattern revert to that of the original cell line. Thus, after 3 months, the cultured pure CD133+ population developed a CD133+/CD133− split of 70%/30% respectively. These data are consistent with other studies showing that tumours derived from CD133+ populations ultimately become bimodal. However, it is uncertain why the CD133+ population did not revert to 40% as seen in the original SW480 cell line.

The cultured pure CD133− population developed a stable CD133+ population of around 17%. The study by O'Brien found that CD133− sorted populations could induce tumours containing a CD133+ population and attributed this to contamination of the CD133− cells by CD133+ cells. We think that contamination is unlikely to be an explanation in our case as our time-course studies did not show any difference in rates of proliferation between CD133+ and CD133− cells. Another possibility is that CD133 was re-induced in these cells. Since CD133 mRNA was detected in the CD133− cells, it shows that a low level of transcriptional activity may still have been occurring in cells which were negative for protein expression. This would fit with data from animal studies demonstrating that transit amplifying cells (which lie beyond the stem cell compartment) can re-acquire stem cell properties in the small intestine under appropriate conditions [32]. If this is the case, then it would question the existence of CSCs since, by extension, non-stem cells within a tumour could theoretically acquire stem cell features.

In summary we have shown that there is only one CD133 splice variant expressed in colorectal tissues and there is plasticity of its expression. There is marked variation in the size of the CD133+ population within CRC cell lines although, given that some cell lines have no CD133+ cells and CD133− cells from bimodal populations can be clonogenic, it is unlikely that CD133 is a specific stem cell marker. However CD133 expression is associated with some attributable to stemness and the mechanistic basis of these warrants further investigation.
